# Continuous quality improvement in HIV and TB services at selected healthcare facilities in South Africa

**DOI:** 10.4102/sajhivmed.v22i1.1202

**Published:** 2021-05-12

**Authors:** Sisanda Gaga, Nokuzola Mqoqi, Raymond Chimatira, Singilizwe Moko, Jude O. Igumbor

**Affiliations:** 1 Beyond Zero, East London, South Africa; 2 Centers for Disease Control and Prevention, Pretoria, South Africa; 3 Eastern Cape Provincial Department of Health, Bisho, South Africa; 4 School of Public Health, Faculty of Health Sciences, University of the Witwatersrand, Johannesburg, South Africa

**Keywords:** continuous quality improvement, interventions, HIV/AIDS, services, outcomes, South Africa

## Abstract

**Background:**

Continuous quality improvement (CQI) is essential for HIV and tuberculosis (TB) services. Similarly, a thorough understanding of the requirements and impact of CQI is critical to its successful institutionalisation. However, this is currently lacking.

**Objectives:**

The objective of this study is to describe the CQI implementation process and examine its effect on HIV and TB service delivery at selected primary healthcare facilities in two South African districts.

**Method:**

We used a separate sample, pre- and post-test, quasi-experimental study design based on data collected from the clinical audit of patient cohorts seen in 2014 and 2015 respectively. Quality was measured based on the extent to which prescribed services were provided. Tailored CQI interventions were implemented based on service delivery gaps identified by the 2014 CQI audit. Data were summarised and analysed using a combination of univariate and multivariate analysis.

**Results:**

The services identified as low quality were related to opportunistic infections management and laboratory practices. Compliance to prescribed service items in antiretroviral treatment initiation and monitoring, pharmacy and laboratory management, exceeded 70% across study sites. Over 80% of low quality service delivery items were optimised in less than six months with targeted quality improvement support.

**Conclusion:**

The observed improvements signal the effectiveness of the CQI approach, its capacity to rapidly improve under-performance, its high replicability and the need to provide quality maintenance support to sustain or improve healthcare facilities performing well. The study strongly underscores the need to improve the management of opportunistic infections and complications, particularly TB.

## Background

Continuous quality improvement (CQI) is a management approach used to enhance an organisation’s processes based on its measured performance.^1,2,3^ Continuous quality improvement processes use performance data to inform an iterative and incremental transition towards an optimally performing system by building on successes and improving sub-optimum activities and outputs.^4^

Continuous quality improvement processes are proactive. They are able to identify and remediate latent or future programme challenges and requirements.^5^ The World Health Organization’s (WHO) Health Systems Strengthening (HSS) framework^6^ reemphasises the critical value of CQI models. The framework depicts the quality as a bridge between the building blocks of the healthcare system and their desired outcomes. Thus, an effective HSS model relies on functional CQI processes. This reasoning is in line with the established linkages between CQI implementation and improved health system efficiency, access and outcomes.^7,8,9^ The characteristic value of CQI approaches is their ability to measure process and outcome indicators, with the aim of targeting the implementation of change in the smallest replicable unit within the health system.^10^

The growing number of people living with HIV (PLWHIV) has necessitated the increasing demand for quality care. This means that sub-quality HIV and TB programmes may fail to meet their targets. This scenario and the ‘chronicity’ of the HIV epidemic and its manifestations, could result in the following challenges: insufficient screening of high-risk individuals, failure to adequately link people to care, inability to retain people on treatment, deficiency in re-engaging lost patients, and an increase in patients’ morbidity and mortality. These factors are key drivers of HIV transmission; they increase the costs of care and diminish the programme’s sustainability and outcomes.^11,12^ In order to address these concerns, the systematic approach of CQI models can be applied to yield and optimise the epidemiologic impacts and cost-effectiveness of interventions.^11^

Globally, CQI initiatives have reported varied and remarkable success in the fight against HIV.^13^ Efforts to standardise the HIV programme for CQI processes have, for the most part, been observed in developed countries where the epidemic and context are different from those in Africa.^8,10,11^ The usefulness of CQI processes in low- and middle-income countries (LMICs) remains to be seen, in particular, its potential to accelerate progress towards achieving epidemic control.^1^ Continuous quality improvement was introduced in two districts of South Africa (SA) in 2014 by the Centers for Disease Prevention and Control (CDC), the implementing partner that funded the HIV programmes. Little is known about how the CQI programme was implemented or the success of this intervention to improve the delivery of HIV and TB services. In this research article, we describe the CQI implementation process and examine its effect on the delivery of HIV and TB services at selected primary healthcare (PHC) facilities in two districts of SA.

## Methods

### Study design

A separate sample, pre- and post-test quasi-experimental study design was adopted based on routine programme data from two districts supported by the President’s Emergency Plan for AIDS Relief (PEPFAR) funded HIV/TB programme in SA. The data were collected from the medical records of patients through a retrospective clinical audit of data collected during routine service delivery at two time points: July to December 2014 and July to December 2015. The CQI intervention was implemented for 6 months between the two time points (January–June 2015).

The clinical audits focused on the following five service delivery areas: adult antiretroviral therapy (ART), HIV counselling and testing (HCT), TB case finding and management, and pharmacy and laboratory service delivery areas, which were aligned with various inter-related targets. Such targets are contained in the Joint United Nations Programme on HIV and AIDS (UNAIDS) 90-90-90 strategy,^14^ the district implementation plan (DIP) and in the routine PEPFAR/South African National Department of Health (NDoH) monitoring and evaluation plan. Several questions or items in each service delivery area were designed to identify gaps in the quality of services. The list of questions or items assessed is appended to the document displayed in Appendix 1.

### Study population and sampling

The clinical audits were conducted in 90 supported healthcare facilities in the two districts of SA: District A and District B. Ninety-three per cent of the facilities included were PHC facilities, and the rest of them were community health centres (CHCs). Seventy-one per cent of the healthcare facilities were from District B. About 60% of the facilities were located in rural areas and 40% were in urban areas.

Prior to the implementation of the CQI project in 2014, most of the performance indicators of the health system in both the districts required improvement, as most programme targets were unmet. At the start of the project, District A ranked amongst the 10 worst-performing districts in SA on indicators, such as management of inpatients.^15^ The worst performing indicators for District B included the management of PHC facilities, inpatients, human resources, TB case findings and TB treatment outcomes.^15^

The healthcare facilities included in this evaluation were high-volume sites or those having at least 800 people regularly on ART at the healthcare facility. This is otherwise referred to as total remaining on ART (TROA). High-volume sites are designated by the Department of Health based on the monthly facility headcount, catchment population size, health facility utilisation rate and disease burden. There were 90 study sites purposively selected using the criteria of high HIV or TB burden. All the healthcare facilities with the availability of patients’ folders, in all the five service delivery areas, were eligible for this study. The first cohort consisted of all records of patients initiated on ART from July to December 2014. The second cohort consisted of all records of patients initiated on ART from July to December 2015.

A maximum of 20 patient service records and folders per facility were selected and audited. The 20 folders consisted of the records of TB and/or HIV patients from the five service areas that were assessed (see Appendix Table 1 for the list of items assessed in each service area). A systematic sampling method was used to select the files to be audited. The interval between audited files was calculated based on the total number of eligible files at the site. All available folders were audited for facilities with < 20 folders.

With 20 patient folders selected per healthcare facility, a minimum sample size of 1800 patient folders were audited. The proposed sample size was intended to detect a 95% power and a 5% margin of error. From the sample calculation, it was assumed that there were approximately 72 000 patient folders in the 90 healthcare facilities based on a TROA of about 800. By systematic random sampling, the sampling interval (*k*) of 40 was calculated by dividing TROA (800) by the required sample per facility (20). Using a random starting point (*x*), we were able to establish every *k*th folder to be selected until the number of 20 folders was reached. Incomplete files were replaced with the next *k*th folder.

### Study intervention

The study intervention included the CQI audits and tailored support provided by a roving team of multidisciplinary healthcare providers. The roving CQI audit teams consisted of nurse mentors, information officers, monitoring and evaluation advisors, pharmacy assistants and at least one technical specialist.

The multidisciplinary audit teams provided comprehensive and integrated support to the audited healthcare facilities. The audit teams were trained on CQI processes, reporting protocols and problem remediation mechanisms using a standard operating procedure that was developed for the audits. Clinical practitioners with research and health system strengthening experience provided training to the roving teams. This was performed to ensure quality and implementation consistency amongst all teams.

Each audit involved patient file reviews and scoring by the team. Thereafter, the overall facility performance report was provided to the respective healthcare facility managers. Red flags (bottlenecks), as well as improvement plans, were discussed with the health facility manager. The focus of the intervention was on how to improve the activities and indicator element that was not performing well, that is, < 50% compliance to prescribed service items. Based on identified needs, the CQI plans with specific interventions varied between healthcare facilities. The interventions ranged from activities to improve drug procurement and dispensary procedures to clinical skills development, mentorship and supportive supervision. They also included improvements in monitoring and evaluation, and information utilisation for decision-making, targeted service improvement, service delivery campaigns and community engagement activities, support with patient flow management and human resource management support. The use of tailored interventions to respond to prevailing service delivery gaps during quality improvement has been found to be efficient and effective.^16,17^

### Data collection tool

A service audit tool was used to assess the quality of HIV and TB services provided. The data audit tool was adapted from the standardised data audit tool developed by the NDoH for routine monitoring of health programmes. The audit tool was developed through an extensive consultative process with inclusive multidisciplinary healthcare teams who were selected from participating healthcare facilities before the first audit in July 2014. The finalised tool was pretested in two randomly selected healthcare facilities. These pilot sites were excluded from the main study.

Responses on the tool were coded: 1 = Yes and 0 = No. The number of ‘Yes’ responses, divided by the total number of audit items or questions per service area, determined the total facility score per service area. The formula was adjusted to exclude ‘not applicable’ in the final facility score. Three cut-off points were used to categorise the performance of healthcare facilities in each of the service areas. Green represented facilities that scored ≥ 85%, amber for facilities that scored between 50% and 84%, and red signified poor-performing facilities scoring < 50% in an item measured or service area.

Additionally, health facility capacity and performance indicators were obtained from the District Health Information System (DHIS) database. The collected information included facility aggregates reported as percentages and ratios. The indicators collected from the National Indicator Data Set (NIDS) included health facility utilisation rate, nurse and doctor workloads, number of individuals initiated for treatment prior to and during the audit period, number of patients remaining in care during the period, and patient headcount. The list of health facility capacity and performance indicators is available in Appendix 2. With this additional data, we were able to compare the average facility performance based on the routine DHIS indicator 3 months before (April – July 2014) and 3 months after (August – October 2015). These time points are before and after the implementation of the tailored CQI interventions developed by the CQI team and the respective health facility managers. The additional analysis of NIDS data was to triangulate the findings of the record review, and to explore for possible confounders and explanatory variables of the study outcomes.

### Data analysis

The audit data on HIV-TB services were analysed to describe the overall performance of the healthcare facilities’ programme implementation and service quality in the 2014 and 2015 cohorts. All data were analysed using Stata (version 13.0, StataCorp).

The inter-item correlations and Cronbach’s alpha of the respective scales’ service areas were also calculated to assess the reliability of the audits tools. Apart from the original laboratory services audit tool, the rest of the audit tools were reliable, with Cronbach’s alpha at or exceeding the recommended 0.7 mark (Table 1). The reliability of the laboratory services audit tool was improved by deleting items with low inter-item and squared multiple correlations. This analysis was conducted before basic descriptive analysis to determine whether we can create reliable measurement scales using service area quality items.

**TABLE 1:** Reliability of audit tools used to assess the quality of HIV and tuberculosis services.

Service area audit tools	Number of items	Cronbach’s alpha
Adult HIV treatment	15	0.8
HIV counselling and testing	5	0.8
TB case finding and management	10	0.9
Pharmacy	15	0.7
Laboratory (original)	8	0.5
Laboratory (revised)	5	0.7

Inter-item and squared multiple correlation analyses are used to explain the extent to which the performance of one item on a scale is affected by the scores of other items in the respective scales. Therefore, we used this analysis to identify items, the presence or absence of which were affected by the combined presence or absence of the rest of the items in the respective tools. Meaning, if a quality requirement (item) is met, it is highly likely that the rest of the quality requirements (items) in the audit tool are met. The items also had the highest loading in the principal component analysis and communality. We further carried out stepwise regression analysis to identify the strongest predictors of the particular item. We used this analytical approach to identify possible precursors and covariates to target during routine quality maintenance audits with fewer items. Factor analysis was performed to assess the validity of the tools and to explore the possibility of reducing each service area’s tool to fewer clinically and statistically significant questions or items that can be used routinely.

After establishing the validity of the items and scales, we used descriptive analysis in the form of counts and percentages to present variables collected from the clinical audit and NIDS indicators. Differences in cohorts were determined using chi-square or Fisher’s exact test for sparse data. Correlations were performed between the extent of programme implementation and the quality of services provided at the two time points. Using linear regression analysis, the quality improvement measures and categories were adjusted against standard health systems performance indicators from NIDS of their respective service areas. The facility performance in 2014 and 2015 cohorts was compared using chi-square tests for categorical outcomes. Continuous variables were compared with either Mann–Whitney *U*-test or analysis of variance (ANOVA) test. We also calculated comparisons between districts, PHC facilities and CHCs.

### Ethical considerations

Ethics approval to conduct the study was obtained from the University of the Witwatersrand’s Human Research Ethics Committee (No. M161025) and from the Associate Director of Science in the Centre of Global Health, CDC. We conducted a secondary analysis of anonymised data that did not require individual patient consent. Consequently, our ethics approval was a waiver to use secondary data sources. This research article presents aggregate and summary data of all participants, and hence, consent to publish is not required.

## Results

### Service area audit scores

Services areas, such as pharmacy and HCT, reported the highest scores in the 2014 and 2015 audits, whereas TB case finding and management recorded the lowest quality scores in both years, showing a marginal improvement in 2015 (Table 2). The highest percentage differences in the two audits were recorded for the HCT (9%) and laboratory service areas (9%). Table 2 further shows that in 2015, District A reported the highest HCT and adult HIV quality scores. In the same year, the highest pharmacy score was recorded in District B, and TB case finding and management quality audit scores were relatively low in District A (66.7%) and District B (65.2%). Concurrently, District B retained the highest pharmacy score (89.8%), and District A retained the highest adult ART score (84.3%). The highest improvement was reported in laboratory indices in District B (12.2%). The differences in cohorts were considered to be statistically significant (*p* < 0.001), except for District A where a marginal improvement was observed because of its good performance in the previous audit.

**TABLE 2:** Percentage performance in the service areas by districts and cohorts.

Service area	District A		District B		Both districts	
	2014	2015	2014	2015	2014	2015
Adult ART services	84.3	87.5	79.6	84.1	80.0	83.9
HIV counselling and testing	89.8	92.2	82.6	86.9	80.8	89.8
TB case findings	66.7	73.8	65.2	69.2	68.9	72.4
Laboratory services	80.4	86.8	72.4	84.6	77.0	85.6
Pharmacy services	87.1	85.7	89.8	92.6	86.8	89.9

ART, antiretroviral therapy; TB, tuberculosis.

**FIGURE 1:**
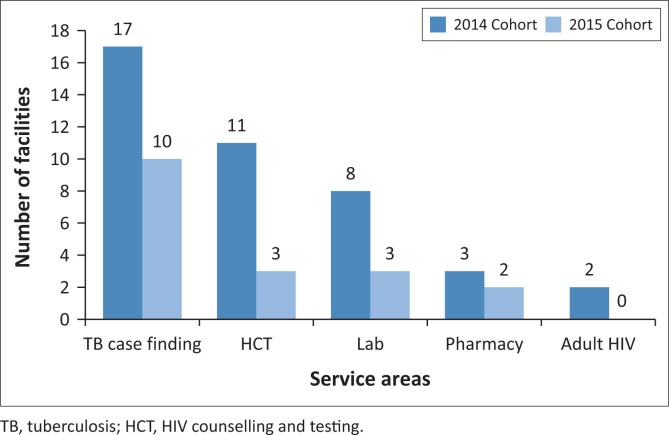
Number of healthcare facilities with red-flagged service areas following the 2014 and 2015 audits.

In 2015, fewer (*n* = 18) healthcare facilities were red flagged for intensive quality improvement in all the service areas compared with those red flagged in 2014 (*n* = 41; Figure 1). The quality audit scores for the red-flagged facilities were < 50% in the respective service areas.

### Best and worst performing items

Table 3 shows the worst and best performing items in the TB case finding and management service area based on the 2014 audit. Tuberculosis case findings recorded the highest number of quality items that attained low audit scores. The performance of the respective service areas represents general improvements in 2015. This observation is against the backdrop that the TB service area has recorded relatively low overall quality scores.

**TABLE 3:** Best and worst performing items in the tuberculosis case finding and management and adult antiretroviral therapy service areas.

Items	Districts	
	A (%)	B (%)
Line probe assay performed for non-converters	27.6	30.4
Baseline AFB tests result TAT under 48 h	28.7	13.9
Client investigated at 11 weeks	37.3	59.5
GeneXpert® result received under 48 h	37.6	15.1
IPT offered to eligible contacts	38.8	32.9
CrAg performed amongst eligible before ART initiated	44.5	57.9
Patient positive for TB symptoms had appropriate investigations ordered	52.6	34.2
Client diagnosed with GeneXpert®	97.0	95.1
Screened patient recorded at the last visit	97.1	89.5
Patient on TDF, AZT, LPV/r or an NVP-based regimen	99.6	99.4

AFB, acid fast bacillus; TAT, turnaround time; IPT, isoniazid prevention therapy; ART, antiretroviral therapy; CrAg, cryptococcal antigen; TB, tuberculosis; TDF, tenofovir disoproxil fumarate; AZT, azidothymidine; LPV/r, lopinavir or ritonavir; NVP, nevirapine.

Items measuring the TB laboratory test turnaround time (TAT) recorded the lowest scores, particularly in District B. The provision of line probe assay (LPA) to non-converters and Isoniazid (INH) Prevention Therapy (IPT) to eligible contacts was consistently low in both districts. Investigation of conversion just before 3 months (at 11 weeks) in line with TB guidelines was relatively poor in both the districts.

The cryptococcal antigen (CrAg) testing item of the adult ART service areas was equally poor in both districts. The low proportion of patients positive for TB symptoms with appropriate further investigation was of concern under the adult ART service area. However, the adult ART service area reported relatively high overall quality audit scores compared with all other service areas. A relatively low documentation of lost or rejected specimens was observed in the performance of laboratory service area of District A.

### Urban–Rural differences in service area audit scores

**FIGURE 2:**
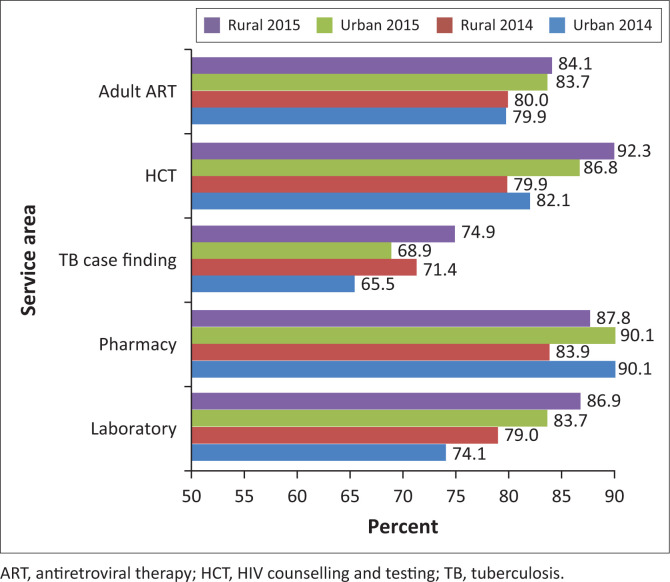
Urban and rural quality audit scores in 2014 and 2015 (pre- and post-continuous quality improvement interventions).

When compared with the urban healthcare facilities, the rural healthcare facilities consistently performed better in raw audit scores for the TB case finding and management and laboratory service areas in both the 2014 and 2015 audits (Figure 2). Notwithstanding the apparent similarity in the performance of urban and rural healthcare facilities at both audits in the adult ART service area, rural healthcare facilities performed slightly better. The respective locations, however, recorded significant improvements in the 2015 cohort (*p* < 0.001). The urban healthcare facilities performed better in the pharmacy service area. The highest improvement (12%) was recorded by rural health facilities in the HCT area. A notable improvement was observed in both the urban and rural areas for the laboratory service area, with a statistical difference observed in 2015 in both locations compared with their performance in the 2014 audit (*p* < 0.001).

### Predictors of items with the highest loading in principal component analysis

The strength of the loading in principal component analysis is an indication of the relationship between a variable and items in the scale or, in this case, the service areas.^18^ The adult ART service area audit item – ‘patient screened negative for any TB symptom and initiated for INH’ – was a predictor of patients remaining on ART 6 months after treatment initiation (*R*^*2*^ = 0.5). The predictors of received laboratory result recorded in the shipping list/specimen book included the following items: ‘rejected or lost results documented’, and ‘facility documenting samples on daily basis in shipping list/specimen book’ (*R*^*2*^ = 0.5). More predictions can be found in the tabular form (Table 4).

**TABLE 4:** Quality assessment items with the highest squared multiple correlation in each service area using the July 2014 baseline audit data.

Audit tools	Highest loading items and their predictors		Multiple correlation*	Communalities %
Adult HIV	Patient who screened positive for TB symptoms had appropriate investigations ordered		0.8	0.9
	Main predictor	Patient screened and recorded at the last visit (*R*^*2*^ = 0.7)		
HCT	HIV + TB patient started on ART?		0.6	0.8
	Main predictors: (*R*^*2*^ = 0.5)	HIV + TB patient enrolled into HIV care		
		HIV + TB patient taking cotrimoxazole prophylaxis		
TB case findings and management	Was client investigated at 11 weeks?		0.8	0.8
	Main predictor	Was client investigated at 7 weeks (*R*^*2*^ = 0.5)?		
Pharmacy	Is the information on the bin card maintained and updated for HIV and TB?		-	0.9
	Main predictors: (*R*^*2*^ = 0.4)	Bin card for each item in storeroom		
		ART in stock		
		Is stock kept on shelves or pallets?		
Laboratory	Are rejected and lost samples documented in the shipping list/specimen book?		0.6	0.8
	Main predictor	Result received recorded in the shipping list/specimen book? (*R*^*2*^ = 0.5)		

TB, tuberculosis; HCT, HIV counselling and testing; ART, antiretroviral therapy.

* , Multiple correlation, this is how well a variable can be predicted by other variables.^19^

%, Communalities, ‘proportion of each variable variance that can be explained by the factors’.^20^

### Performance of selected national indicator data sets

Based on the DHIS data, the 90 audited facilities varied in capacity and performance. District A reported a high healthcare facility utilisation rate, nurse workload and performed well in terms of HIV testing coverage (Table 5). The table also depicts the relatively high HIV prevalence rate in District B, despite the relatively low HIV testing coverage.

**TABLE 5:** Average district capacity and performance as of July 2014 based on national indicator data set.

Capacity and performance indicators*	District A		District B	
	Mean	SD	Mean	SD
PHC utilisation rate (annualised)	3.4	0.4	2.6	0.2
PHC professional nurse clinical workload	53.2	13.2	26.5	12.9
PHC doctor clinical workload	24.5	9.2	51.3	31.3
HIV testing coverage (annualised)	58.2	19.2	34.0	32.3
HIV prevalence amongst clients tested	2.4	2.5	8.6	6.6

PHC, primary healthcare; SD, standard deviation.

* , Indicator definitions and national averages are available in the District Health Barometer 2014–2015.^15^

**FIGURE 3:**
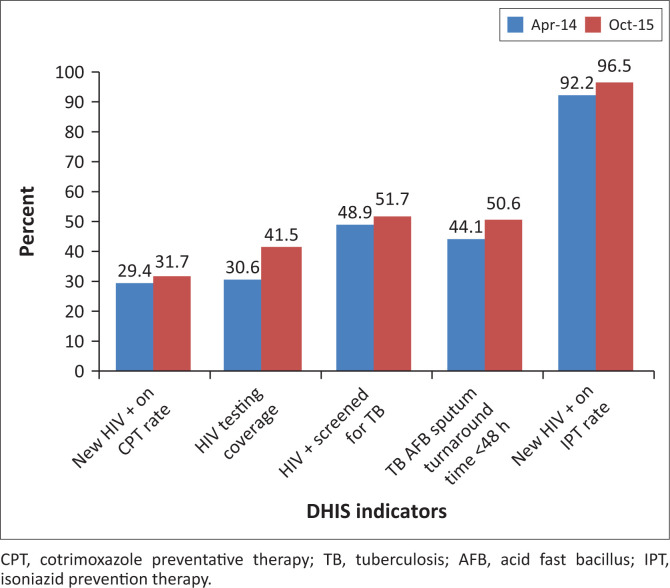
Performance of facilities by district health information system indicator pre-and post-continuous quality improvement intervention (April 2014 and October 2015).

The average facility performance based on routine DHIS indicators in the 3 months before (April – July 2014) and 3 months after (August – October 2015) CQI implementation was compared (Figure 3). These time points are before and after the implementation of the tailored CQI interventions. The highest improvements were observed in HIV testing coverage (11%) and TB acid fast bacillus (AFB) sputum results turnaround time of < 48 h (6%). The observed differences were also statistically significant (*p* < 0.001). In addition to information shown in Figure 3, the data revealed that TB case findings significantly increased by approximately 50% from 1.9 to 2.8 (*p* < 0.001).

### Relationship between service area quality scores and national indicator data set

Weak to moderate associations existed between service area quality scores and the national indicator data set (Table 6). The strongest correlation was observed between the TB case finding and management audit score and the NIDS, measuring the proportion of HIV-positive patients screened for TB rates. Similarly, there were positive associations between the HCT service area score and the NIDS indicator, measuring the proportion of HIV-positive patients screened for TB and HIV testing coverage. The prevalence rate of HIV reported in the NIDS consistently demonstrated a negative relationship with the service areas quality scores to the extent that the higher the HIV prevalence rate the lower the quality scores. The laboratory service areas quality score showed associations with more NIDS indicators. Healthcare facilities with a higher utilisation rate and workload performed better in the laboratory service area, whilst healthcare facilities with a higher HIV prevalence and higher TB screening rate performed poorly in the laboratory service area.

**TABLE 6:** Correlations between service area scores and routinely collected national indicator data set.

National indicator data set	Service area correlation coefficients				
	Adult ART	HCT	TB case findings and management	Pharmacy	Laboratory
Median HIV testing coverage (April – July 2014)	**0.27**	**0.24**	0.14	0.05	**0.27**
Median HIV prevalence rate (April – July 2014)	**−0.27**	**−0.26**	−0.09	0.01	**−0.24**
Median HIV + patient screen for TB rate (April – July 2014)	0.10	**0.28**	**−0.45**	**0.24**	**−0.19**
Median HIV + initiated on IPT rate (April – July 2014)	0.13	0.16	0.15	0.10	**−0.14**
Median utilisation rate (April – July 2014)	0.03	0.19	0.16	−0.02	**0.27**
Median nurse workload (April – July 2014)	0.04	0.08	0.03	−0.02	**0.25**
Median PHC doctor workload (April – July 2014)	−0.15	−0.11	**−0.43**	0.02	−0.07

Note: Correlations significant at 0.01 level (two-tailed) is in bold.

ART, antiretroviral therapy; HCT, HIV counselling and testing; TB, tuberculosis; IPT, isoniazid prevention therapy; PHC, primary healthcare.

Table 6 also suggests that healthcare facilities that performed better in the TB case management (in terms of the service area score) might not have performed very well with the screening of new HIV-positive patients for TB (and vice versa). The facilities with better TB case management also had a lower doctor workload.

**FIGURE 4:**
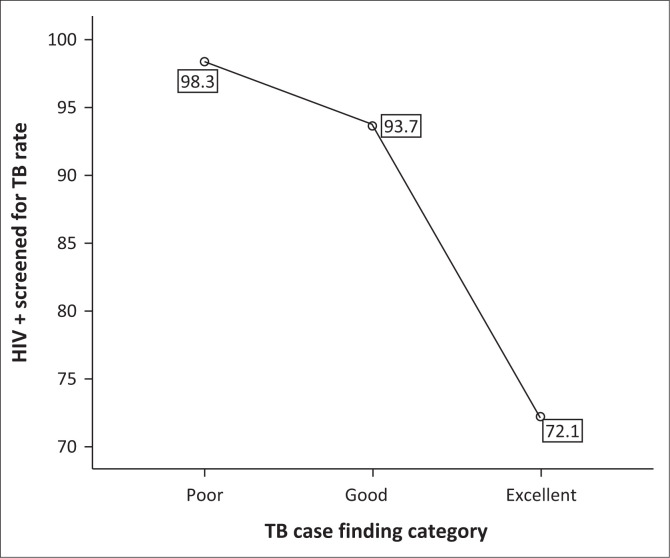
Average HIV + patient screened for tuberculosis rate by the tuberculosis case finding and management service area quality category.

Figure 4 shows the correlation coefficients of the average HIV-positive patient screened for TB rates and the TB case finding and management categories in both years and districts. Healthcare facilities that were performing relatively better than the other healthcare facilities in the TB case finding and management service area reported significantly less HIV-positive patients screened for TB rate compared with those in the poor and good categories.

## Discussion

This report provides evidence of improvements in processes and outcomes in all service areas following the CQI intervention. This observation is informed by the significant decline in the number of healthcare facilities with red-flagged audit scores (< 50% compliance with prescribed services), as well as the observed improvements in most service areas across districts amongst the 2015 cohort in comparison with the 2014 cohort.

Whilst marginal improvements may be observed in scores aggregated in both the districts, the magnitude of effect varies across service areas and districts. This may be attributed to the varying demand or supply ratios (e.g. patient load vs. human resources) across facilities and service areas, as well as other factors unique to service areas, districts and facilities.

Furthermore, the inverse relationship observed between the quality and volume of services provided suggests that the poor quality observed in some healthcare facilities may be because of a high volume of work. Consequently, increasing the supply and efficiency of human resources may improve the quality scores in such facilities. Further research is, however, needed to explore this relationship.

A marked improvement was observed in HCT and laboratory samples and test results management. This may be because of implementing partner’s generic interventions to improve both components at all its supported sites. Implementing partner’s focus on these service areas is over and above the site-specific interventions that were developed and implemented during the CQI project.

During this assessment, it was found that the HCT recorded the most marked reduction in the number of facilities red flagged between the 2014 and 2015 audits. A positive association observed between quality scores for HCT service area and NIDS indicators related to HIV and TB supports other research studies and policy documents, which suggest that a synergistic approach to HIV or TB management will result in better service outcomes.^21^

The integration of these services is, however, not without systemic challenges. Insufficient stakeholder consultations, poor leadership and political will are common bottlenecks to the implementation of integrated HIV and TB policy in parts of SA.^22^ A similar study carried out in Uganda reported integration constraints in addition to other factors, such as poor planning and coordination, as well as inadequate provider knowledge of interpreting TB laboratory results.^23^ The health system governance bottlenecks may account for the differential improvement rates observed, particularly in District A, which had the most marked quality improvement audit scores in this service area.

Interestingly, whilst both districts scored high with respect to ensuring that patients are diagnosed with the GeneXpert®^24^ system, ensuring a 48-h turnaround time for the results was suboptimal. The implication is that whereas clients may be getting the needed services, there is a need to further improve implementation fidelity of service processes. Even though the GeneXpert® test for TB is reputed to deliver results in 2 h under ideal conditions, operational barriers commonly affect this turnaround time in real-world settings.

Piatek et al.^24^ identified the following barriers: inadequate human resources, practices of batching specimens, and inefficient specimen referral and transport networks. Thus, addressing these issues as part of this CQI initiative may improve the quality of GeneXpert® services, including the turnaround time.

We also found that healthcare facilities with high quality scores for the TB case finding service area also had lower smear positive rates. This may imply that available healthcare workers were unable to meet the demand for screening services, and thus, there were trade-offs between the quality of services and meeting the quantity of demand for services at the healthcare facilities. These trade-offs are not unique to the South African context. A recent study in the neighbouring country Lesotho also identified inadequate workforce as a major reason for poor adherence to TB control guidelines.^25^ The observed pattern could be because of the discretionary power of frontline health workers in determining how to implement the guidelines and policies.^26^ In this scenario, the decision to trade quality of services for volume is likely to result from reactionary discretion of the healthcare workers, when faced with work overload, irrespective of policies and guidelines. Walker and Gilson^27^ studied the attitudes of frontline nurses in SA, and found that personal views and values influence healthcare workers’ discretion to adhere to policies and guidelines. Thus, in order to minimise the impact of frontline discretionary power on quality-of-service delivery, efforts should be made to ensure adequate distribution of healthcare workers. Furthermore, healthcare workers require continuous and/or ongoing training on guidelines, with emphasis on the importance of adherence to quality. Enhanced supportive supervision may also limit the discretionary space of frontline healthcare workers.

Specific activities that require further attention include ensuring that line probe assays are performed for non-converters, IPT is initiated for eligible contacts, and the turnaround time for AFB and GeneXpert® results is improved.

### Limitations of the study

Whilst this research study provides useful insights into the effect of a CQI process in enhancing the delivery of HIV and TB services in parts of SA, notable study limitations should be highlighted. The quality of care reported in the study did not include patient valuation of services provided despite its importance in quality measurement. However, we focused on the process of care delivery, which is one of the intermediaries of the six elements of care improvement proposed by the Institute of Medicine.^28^ The six elements included patient-centred care and satisfaction, timeliness, safety, equity, efficiency and effectiveness. We did not compare our study sites with non-intervention sites to fully substantiate the impact and ascertain the efficacy of the intervention or if it translated to patient health outcomes. Nonetheless, the short duration of the intervention, the high number of healthcare facilities covered and the significant improvement in districts with a long history of poor performance^15^ may give credence to our CQI intervention. The study’s heavy reliance on routine health services data that are prone to incompleteness should also be noted. Furthermore, the study did not measure the long-term durability and sustainability of the CQI process. However, this investigation strongly demonstrates the extent to which intended services are provided. Such information is essential to gauge and promote adherence to evidence-based clinical guidelines whilst relying on appropriate measures to address other limitations.

### Conclusion and recommendations

This research study revealed overall improvement in the quality of adult ART services between 2014 and 2015 in both districts. The adult ART service area had relatively high overall quality scores compared with all the other service areas. However, whereas quality scores were very high with respect to screening and treatment services, more attention should be paid to improving screening for opportunistic infections, such as CrAg, as well as strengthening clinical integration of TB or HIV services. For example, a significant proportion of eligible clients did not have CrAg performed before the commencement of ART. Whilst the cause of this observation may be beyond the scope of this study, a study in the Western Cape singled out forgetfulness to order the test by providers as the major cause of this implementation gap.^29^ Other authors have recommended a reflex laboratory testing approach as a more effective alternative to provider-induced testing. Reflexed tests automatically result in the order of one or more secondary tests based on predetermined criteria applied to the primary test.^30^ Ultimately, targeting and improving poor performing items could improve any service area’s overall quality score.

This research study contributes to empirical evidence of the effectiveness of the CQI intervention on service delivery processes and outcomes in SA. Our claim stands on the significant improvement in service area outcomes following our CQI intervention. Various types of CQI methods have been widely adopted in healthcare with numerous reports of success.^8^ This assessment corroborates existing studies, which found the use of CQI both feasible and acceptable with respect to HIV or TB case findings and management.^22^

It has also been reported, elsewhere, that the success of CQI initiatives depends on frontline health workers’ involvement, as well as strong organisational support.^19^ Therefore, we recommend adequate capacitation and distribution of healthcare workers to match the demand for services. Strategies, such as improving supportive supervision of health workers at service delivery points and strengthening clinical governance, will ensure compliance with service delivery guidelines and enhance positive organisational behaviour. The adoption of available technological solutions to help to minimise errors may also improve quality and human resource efficiency. Finally, strengthening integrated service delivery, particularly the TB and HIV interphase, should be prioritised to promote human and material resource efficiency.

## Appendix 1

**TABLE 1-A1:** List of items used for quality of care audit.

S/N	Items
**Facility adult HIV treatment**	
1	Is baseline CD4 count recorded?
2	Is baseline WHO clinical stage recorded?
3	Is the patient’s WHO clinical stage recorded at the last visit?
4	Is the patient’s weight recorded at the last visit?
5	Is the patient eligible for CrAg test at ART initiation (CD4 < 100)?
5.1	If the patient is eligible, was CrAg performed before ART was initiated?
6	Is the patient on a TDF, AZT, LPV/r (Alluvia) or NVP-containing regimen?
6.1	If yes, is creatinine, HB, ALT, cholesterol or triglyceride done at baseline and at follow-up visits as per national guidelines?
7	Was the patient’s TB screening documented at the last visit?
8	Did the patient screen positive for any TB symptoms?
8.1	If the patient screened positive for TB symptoms, were appropriate investigations ordered for PTB or EPTB?
9	If the patient screened negative for any TB symptoms and is eligible for IPT, was the patient initiated on isoniazid?
10	Is the patient eligible for CPT, i.e. WHO 2, 3 or 4; and/or CD4 < 200?
11	If the patient is eligible, was cotrimoxazole (or with contra-indications, dapsone) initiated?
12	Is this patient still on ART after 6 months of ART initiation?
**HCT TB – HIV integration service**	
13	Are TB patients (new and relapsed) with an HIV status recorded (*on both TB register and blue card*)?
14	Is the HIV-positive TB patient *enrolled into HIV* care (SCR opened, CD4 recorded)?
15	Is the HIV-positive TB patient *taking cotrimoxazole (CPT)*?
16	Is the HIV-positive TB patient started on ART?
17	Is the HIV-positive TB patient started on ART *during the first 2 to 8 weeks* of TB treatment?
	Age – At first diagnosis
	DOB – At first diagnosis
	Sex – At first diagnosis
**TB services and case finding**	
18	Was the client diagnosed on GXP?
19	Is the GXP result received in less than 48 hours?
20	Is baseline smear AFB done for eligible client?
21	Is baseline smear AFB result TAT within or < 48 hours?
22	Is the TAT correctly documented in the case identification register?
23	Was the correct coding done according to the type of TB?
24	Is the patient appearing on the TB Diary?
25	Was the patient investigated at 7 weeks (smears for AFB taken)?
26	Did the patient smear convert at 7 weeks?
27	Was LPA done for non-converters?
28	Was the patient investigated at 11 weeks (smears for AFB taken)?
29	Was 2nd and/or 3rd smear taken?
29.1	If yes, are results recorded in the register?
30	Is the patient’s outcome recorded in the register?
30.1	Is the outcome correct?
31	Was TB contact identification and tracing done?
31.1	Were the contacts screened?
31.2	Was IPT offered to eligible contacts?
**Facility pharmacy services**	
32	Is there a bin card for each item in the storeroom or electronic system (either)?
33	Is the information on the bin card maintained and updated for HIV and TB?
	ARVs
	Co-trimoxazole
	Isoniazid
	TB medication
34	Is the stockroom temperature monitored (information on the chart)?
35	Is the air conditioner working?
36	Is the temperature less than 25°C?
37	Is the fridge temperature monitored?

WHO, World Health Organization; CrAg, Cryptococcal Antigen; ART, antiretroviral treatment; TDF, tenofovir disoproxil fumarate; AZT, azidothymidine; LPV/r, lopinavir or ritonavir; NVP, nevirapine; HB, haemoglobin test; ALT, alanine aminotransferase; TB, tuberculosis; PTB, pulmonary tuberculosis; EPTB, extra-pulmonary tuberculosis; IPT, isoniazid preventive therapy; INH, isoniazid; CPT, cotrimoxazole preventative therapy; HCT, HIV counselling and testing; SCR, serum creatinine; DOB, date of birth, GXP, GeneXpert; AFB, acid fast bacillus; TAT, turnaround time; LPA, Line Probe Assay; ARVs, antiretrovirals.

## Appendix 2

### List of indicators and data requested from the National Department of Health

All data requested are health facility level aggregate data and has no patient unique data. They include:

1. HIV prevalence
2. Health facility utilisation rate
3. Nurse and doctor workloads
4. Number of people initiated on treatment during the audit period
5. Number of patients remaining in care during the period
6. Patient headcount during the audit period
7. Number of HIV and TB deaths reported during the audit period
